# Psychological distress during the COVID-19 pandemic in Canada

**DOI:** 10.1371/journal.pone.0277238

**Published:** 2022-11-17

**Authors:** Roland Pongou, Bright Opoku Ahinkorah, Stéphanie Maltais, Marie Christelle Mabeu, Arunika Agarwal, Sanni Yaya

**Affiliations:** 1 Department of Economics, Faculty of Social Sciences, University of Ottawa, Ottawa, Canada; 2 REMS Consult Limited, Sekondi Takoradi, Western Region, Ghana; 3 School of Public Health, Faculty of Health, University of Technology Sydney, Sydney, Australia; 4 School of International Development and Global Studies, University of Ottawa, Ottawa, Canada; 5 Department of Economics, Stanford University, Stanford, California, United States of America; 6 Department of Global Health and Population, Harvard T.H. Chan School of Public Health, Boston, Massachusetts, United States of America; 7 The George Institute for Global Health, Imperial College London, London, United Kingdom; Teesside University, UNITED KINGDOM

## Abstract

**Background:**

During major pandemics such as COVID-19, the fear of being infected, uncertain prognoses, and the imposition of restrictions may result in greater odds of emotional and psychological distress. Hence, the present study examines the predictors of psychological distress during the COVID-19 pandemic in Canada, and how they differ by gender.

**Methods:**

Data of 2,756 adults aged 18 years and above from a cross-sectional online survey conducted between July and October 2020 was used for this study. A multivariable logistic regression analysis was carried out. The results were presented as adjusted odds ratio (aOR) with their respective confidence interval (CI).

**Results:**

Lower odds of psychological distress were found among males compared to females and among individuals aged 45–64 or 65–84 years compared to those aged 18–44. The odds of psychological distress decreased with a rise in income, with individuals whose annual income was greater than or equal to $100,000 being less likely to experience psychological distress compared to those whose income was less than $20,000. The odds of psychological distress were higher among residents of Ontario compared to residents of Quebec. Similarly, the odds of psychological distress were higher among individuals who reported experiencing COVID-19 symptoms compared to those who did not report any COVID-19 symptoms. The disaggregated results by gender showed that age, province, and self-reported COVID-19 symptoms had significant associations with psychological distress in both males and females, but these effects were more pronounced among females compared to males. In addition, income was negatively associated with psychological distress for both males and females, with this effect being stronger among males.

**Conclusion:**

Five exposure variables (gender, age, province, experiencing COVID-19 symptoms, and total annual income in 2019) significantly predicted the likelihood of reporting psychological distress during the COVID-19 pandemic in Canada. Clearly, there is an imminent need to provide mental health support services to vulnerable groups. Additionally, interventions and policies aimed at combating psychological distress during pandemics such as COVID-19 should be gender specific.

## Introduction

On March 11, 2020, the World Health Organisation (WHO) declared the novel Coronavirus-2019, hereinafter referred to as COVID-19, a global pandemic [1]. Subsequently, different countries across the globe instituted several preventive and control mechanisms to curb the spread of the virus among the population. Notable among these strategies include the imposition of restrictions on movement, closure of schools and non-essential businesses, and mandatory quarantines, social distancing, and isolation of infected persons [2]. By the end of March 2020, 136 countries had implemented one or a combination of the aforementioned strategies [3]. Soon, the WHO also outlined some preventive measures to control the spread of the virus which included regular handwashing, the use of alcohol-based hand sanitizers, physical distancing, and the use of personal protective equipment (PPE) such as face masks [4].

In Canada, the first case of community spread of COVID-19 was reported in March 2020, nearly two months after the very first confirmed case of the infection [5]. Thereafter, the various provincial governments implemented stringent measures against the spread of the virus among communities. For example, in Ontario, there was the closure of schools and non-essential businesses, theatres, indoor recreational programmes, parks, and public libraries [6]. Analogously, Quebec imposed mandatory social distancing and isolation of infected persons, as well as closed schools and non-essential businesses [7]. Despite the fact that these preventive and stringent control measures are vital for reducing the incidence of the disease among the population, they are likely to have some negative effects on the mental health of exposed individuals.

During major pandemics such as COVID-19, the fear of being infected, uncertain prognoses, impositions of restrictions that infringe on the freedoms of people, are likely to result in greater odds of emotional and psychological distress [8]. Published studies have documented various mental health challenges experienced during the COVID-19 pandemic in several countries including China [9], the United States [10] and the United Kingdom [11]. For instance, in a recent review by Brooks et al. [12], the authors posit that COVID-19 is associated with several psychological distresses including anger, anxiety, fear, depression, and insomnia. Similarly, Rajkumar [13] also reveals that since the onset of the pandemic, depression and anxiety increased by about 16–28%, and self-reported stress increased by about 8%.

Before the COVID-19 pandemic, it was estimated that at least one in three Canadians would suffer psychological distress and other forms of adverse mental health outcomes during their lifetime, with those in socially and economically marginalised groups having the highest tendency to develop suboptimal mental health [14]. These dynamics have been affected by COVID-19 and are reflected in an increase in the proportion of Canadians with psychological distress. A study reported that 37.4% of Canadians experienced worsened mental health, including psychological distress, because of the COVID-19 pandemic [2]. Thus, it is undeniable that the COVID-19 pandemic is having a significant impact on the mental health of Canadians.

Although there are several studies that shed light on the coping mechanisms that individuals have adopted to mitigate the effects of the COVID-19 pandemic on their overall mental health in Canada [14, 15], there are only a few empirical studies analyzing the relationship between socioeconomic and demographic factors and COVID-19 symptoms on the one hand, and psychological distress on the other hand. Moreover, studies that assess how the determinants of psychological distress during the COVID-19 pandemic varied by gender are limited. Hence, using evidence from a national cross-sectional online survey in Canada, the present study examines the main predictors of psychological distress, with a particular focus on the impact of self-reported COVID-19 symptoms. In addition, this study examines how these predictors vary by gender.

## Materials and methods

### Study design and data collection

We conducted an online survey to collect data using the SurveyMonkey platform. This survey was cross-sectional in nature and adapted from a study by Canning et al. in the US [16]. Permission was obtained to use and modify the questionnaire (S1 File). All data underlying the findings described in this paper are fully available without restriction. Although this survey comprises three waves of data collection, for the present study first wave data, collected between July and October 2020, are used. Data on demographic characteristics, COVID-19-related symptoms, mental health conditions, recent work experiences, and social distancing behaviour were collected from the respondents. Averagely, it took 8 minutes and 7 minutes to complete the francophone and anglophone surveys, respectively. In total, 4,875 responses across Canada were collected in the first wave, with 3,225 responses in English and 1,650 in French. However, since this paper focuses on COVID-19-related mental health issues, only respondents who had completed the mental health module were considered. Hence, a sample size of 2,756 was considered for this study.

### Outcome variable

**Fig 1:**
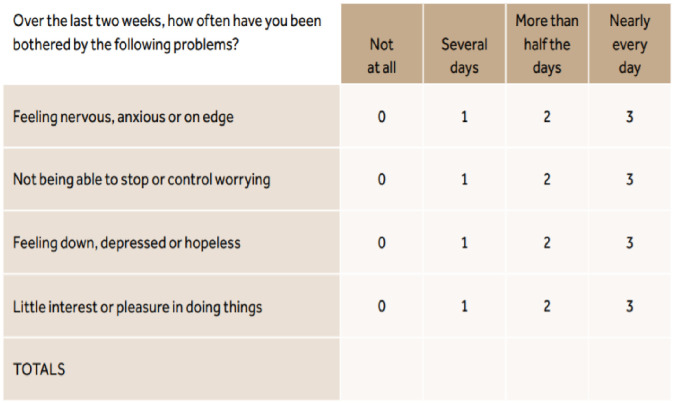
Patient Health Questionnaire-4. Source: Kroenke et al., 2009 [19].

In this study, the outcome variable was psychological distress. To derive this variable, respondents were asked whether they had experienced the following mental issues within the past 2 weeks: (1) feelings of anxiety or feeling nervous; (2) not being able to stop or control worrying; (3) feeling down, depressed, or hopeless; and (4) little interest or pleasure in doing things. Feeling nervous or anxious and being unable to stop worrying were considered an indicator of anxiety, while feeling down, depressed, or hopeless and having little interest or pleasure in doing things were considered an indicator of depression. These questions were derived from the Patient Health Questionnaire-4 (PHQ-4) (Fig 1). The responses were categorised as “not at all”, “several days”, “more than half the days”, and “nearly every day”, with ratings from 0–3 for each statement [17]. Total scores (which can vary from 0 to 12) were rated as normal (0–2), mild (3–5), moderate (6–8), and severe (9–12) psychological distress.

### Exposure variables

The exposure variables in this study were gender (male, female), age (18–44, 45–64, 65–84, and 85 years and above), province (Alberta, British Columbia, Ontario, Quebec, other provinces), race (white, black, mixed race, other race), highest level of education (high school/less, college, postgraduate), total income in 2019 in Canadian dollars (< $20,000, between $20,000—less than $50,000, between $50,000 and <$100,000, $100,000 or more), number of household members (coded as 1, 2–3, and 4 or more), belonging to a minority group, and self-reported COVID-19 symptoms, with each symptom coded as a dichotomous variable indicating whether an individual had the symptom or not.

### Statistical analysis

Descriptive and bivariate analyses were performed using the Stata version 14 software (Stata Corp, College Station, Texas, USA). The descriptive analysis showed the weighted frequency and weighted percentage distribution of the respondents. The bivariate distribution of the categories of psychological distress across the characteristics of the respondents was also performed. Next, the multinomial logit model was used to assess the predictors of psychological distress. Both bivariate and multivariable logit models were estimated. The results were presented using coefficient plots. Finally, the results of the multivariable multinomial logit model were disaggregated by gender and presented using coefficient plots. All statistical significance was obtained at a 95% confidence interval. We followed the Strengthening the Reporting of Observational Studies in Epidemiology (STROBE) reporting guideline in reporting the results. Both descriptive statistics and regression results used weights that were generated using the distributions of gender, age, and province from the Demographic Estimations program at Statistics Canada (StatCan) to have national representativeness. We computed sampling weight for each sex-age-province stratum. Specifically, for each sex-age-province stratum, the weight is equivalent to the ratio of the share (probability of selection) of this stratum in the Census and the share of the same stratum in the survey sample.

### Ethical considerations

The study was approved by the Office of Research Ethics and Integrity at the University of Ottawa prior to the data collection (OREI reference#: S-06-20-5911). Only persons who are residents of Canada and over the age of 18 were eligible to participate in the study. The survey tool had an implied consent that respondents had to read and accept before their participation in the study. All participants’ data were anonymised before analyses. Participation was voluntary and respondents had the right to skip any question that they deemed sensitive to answer.

## Results

### Socioeconomic and demographic characteristics of respondents

Table 1 presents the results of the socioeconomic and demographic characteristics of respondents. More than half of the respondents were female (51.50%). Most of the respondents were aged 18–44 years (46.71%), were living in Ontario (38.97%), were of the White race (85.80%), did not belong to any minority group (87.89%), had college education (65.19%), and had an annual income between $50,000 to less than $100,000 (35.82%). Most of the respondents belonged to a household of 2–3 members (57.29%). Around 17% of the respondents reported experiencing a COVID-19 symptom.

**Table 1:** Descriptive statistics on socioeconomic and demographic characteristics of respondents.

Variables	Weighted Frequency	Weighted Percentage
**Gender**		
Female	1419	51.50
Male	1337	48.50
**Age**		
18–44	1232	46.71
45–64	926	33.62
65–84	541	19.63
85 years and above	56	2.04
**Province**		
Alberta	308	11.16
British Columbia	388	14.08
Ontario	1074	38.97
Quebec	615	22.32
Other provinces	371	13.47
**Race**		
White	2364	85.80
Black	83	3.02
Mixed race	177	6.41
Other race	132	4.78
**Minority group**		
No	2422	87.89
Yes	334	12.11
**Highest level of education**		
High school or less	379	13.76
College	1797	65.19
Postgraduate	580	21.05
**Total income in 2019**		
Less than $20,000	635	23.04
$20,000 to less than $50,000	852	30.90
$50,000 to less than $100,000	987	35.82
$100,000 or more	282	10.25
**Number of household members**		
1	490	18.11
2–3	1550	57.29
4 or more	665	24.60
**Self-reported COVID-19 symptoms**		
No	2291	83.11
Yes	465	16.89
**Total sample size**	**2756**	**100.00**

NB: The category “Other provinces” includes Manitoba, New Brunswick, Newfoundland and Labrador, Northwest territories, Nova Scotia, Nunavut, Prince Edward Island, Saskatchewan and Yukon.

### Distribution of psychological distress in Canada

**Fig 2:**
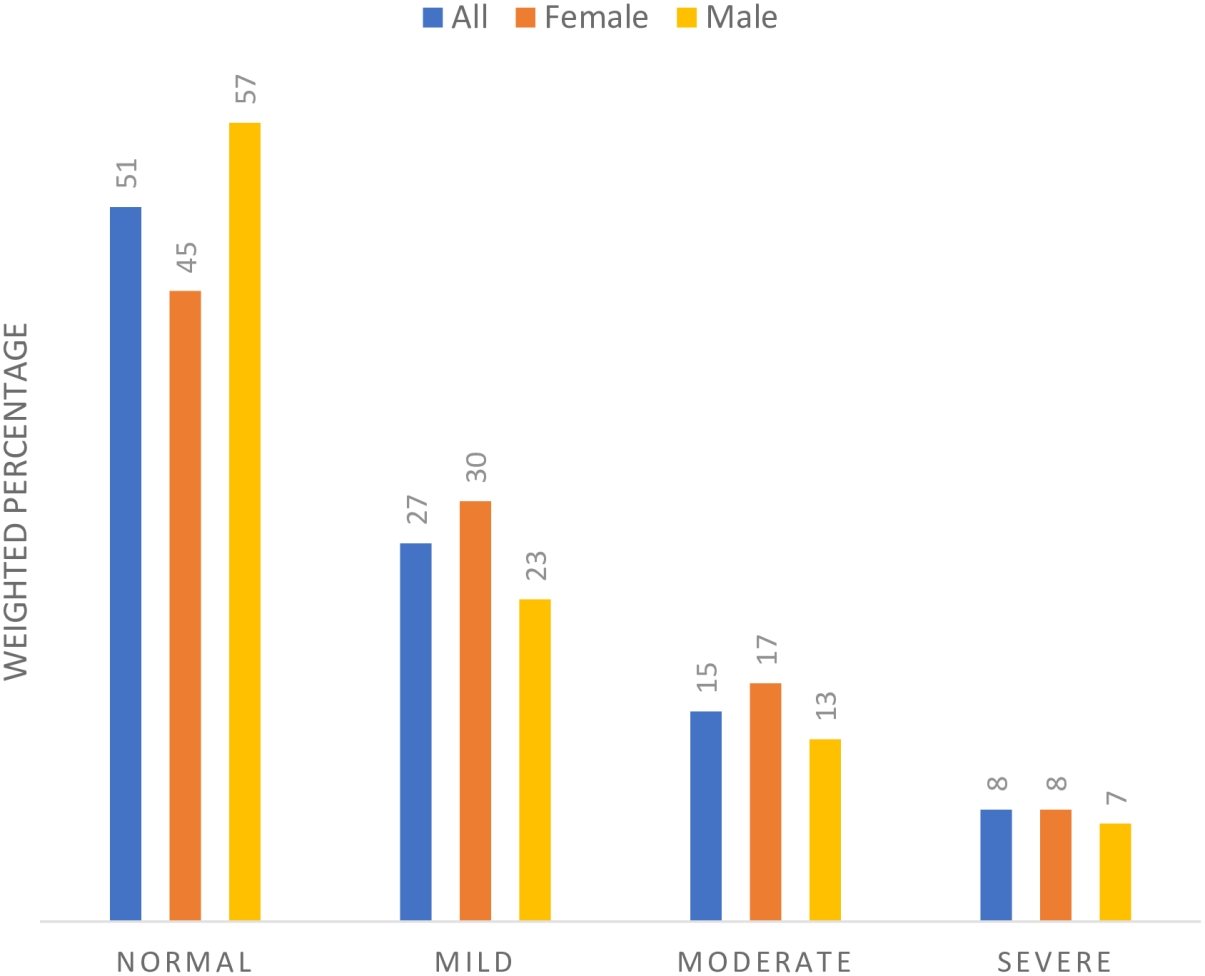
Distribution of psychological distress among respondents and across gender.

Fig 2 shows the distribution of psychological distress scores across gender. It shows that most of the respondents had normal psychological distress scores (51%), followed by those with mild psychological distress scores (27%), and by those with moderate to severe psychological distress scores (23%). More males (57%) than female (45%) had normal psychological distress scores, and more females (8%) than males (7%) had severe psychological distress scores. The results further show variations in the distribution of psychological distress scores across other characteristics of the respondents (Table 2). Table 2 suggests that respondents experiencing COVID-19 symptoms were more likely to experience mild to severe psychological distress than their counterparts with no COVID-19 symptoms. Similarly, respondents under 45 years old, less educated respondents, and those with low income were more likely to report severe psychological distress.

**Table 2:** Distribution of psychological distress across socioeconomic and demographic factors of respondents.

Variables	COVID-19 related psychological distress among respondents				p-values
	Normal	Mild	Moderate	Severe	
**Gender**					p<0.001
Female	45.21	30.09	16.51	8.19	
Male	56.55	23.25	12.85	7.35	
**Age**					p<0.001
18–44	35.49	29.82	22.37	12.32	
45–64	56.22	26.74	11.52	5.52	
65–84	70.82	22.65	4.40	2.13	
85 years and above	100	0	0	0	
**Province**					p<0.001
Alberta	50.52	22.83	18.42	8.24	
British Columbia	50.39	23.76	13.56	12.30	
Ontario	46.63	29.46	16.27	7.64	
Quebec	59.04	26.74	8.61	5.60	
Other provinces	49.21	25.42	18.63	6.74	
**Race**					p<0.05
White	51.83	26.15	14.32	7.70	
Black	46.46	34.97	15.51	3.06	
Mixed race	47.46	32.25	16.04	4.25	
Other race	37.69	25.33	20.02	16.96	
**Minority group**					p = 0.069
No	51.17	26.34	14.67	7.82	
Yes	47.36	29.88	15.19	7.57	
**Highest level of education**					p<0.05
High school or less	49.64	26.89	15.11	8.36	
College	51.98	24.95	14.94	8.14	
Postgraduate	47.49	32.33	13.87	6.31	
**Total income in 2019**					p<0.001
Less than $20,000	36.10	27.21	22.38	14.30	
$20,000 to less than $50,000	51.46	28.50	12.35	7.69	
$50,000 to less than $100,000	56.33	24.81	13.68	5.19	
$100,000 or more	61.64	27.41	8.46	2.50	
**Self-reported COVID-19 symptoms**					p<0.001
No	54.62	26.08	11.97	7.33	
Yes	31.49	30.14	28.34	10.03	
**Number of household members**					p<0.001
1	58.76	20.78	13.59	6.87	
2–3	50.94	27.78	12.90	8.39	
4 or more	43.30	29.29	20.38	7.02	

*p-values obtained from chi-square test

### Multinomial logit results on predictors of COVID-19 related psychological distress

**Fig 3:**
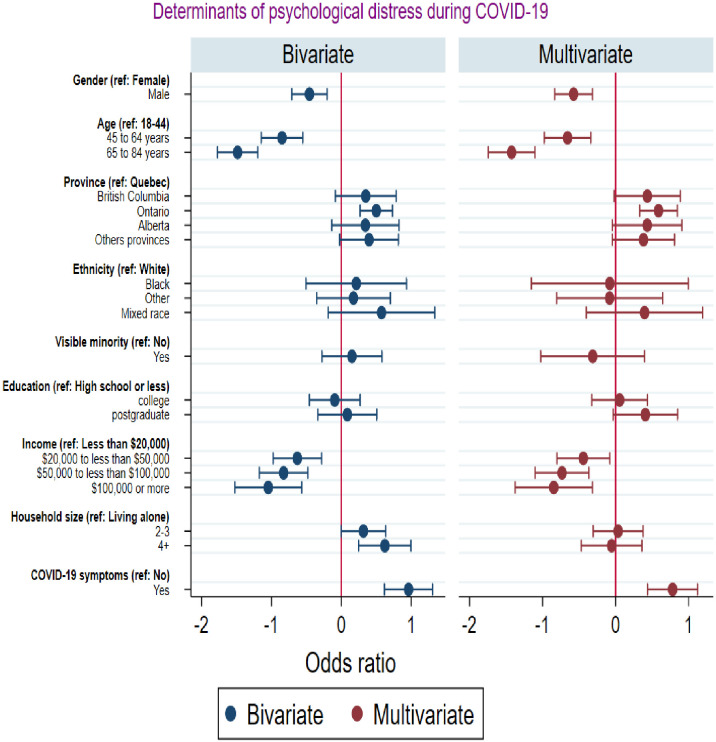
Multinomial logit results on the predictors of psychological distress.

Fig 3 shows the results of the estimation of the multinomial logit models on predictors of psychological distress. At the bivariate level, gender, age, province, income, household size, and self-reported COVID-19 symptoms were significantly associated with psychological distress. The multivariable results show lower odds of psychological distress among males compared to females and among individuals aged 45–64 or 65–84 compared to those aged 18–44. The odds of psychological distress decreased with rising income, with respondents whose annual income was greater than or equal to $100,000 being less likely to experience psychological distress compared to those whose annual income was less than $20,000. The odds of psychological distress were significantly higher among individuals who lived in Ontario compared to those living in Quebec. Strikingly, the odd of psychological distress was significantly higher for respondents who reported COVID-19 symptoms compared to those who did not report COVID-19 symptoms.

**Fig 4:**
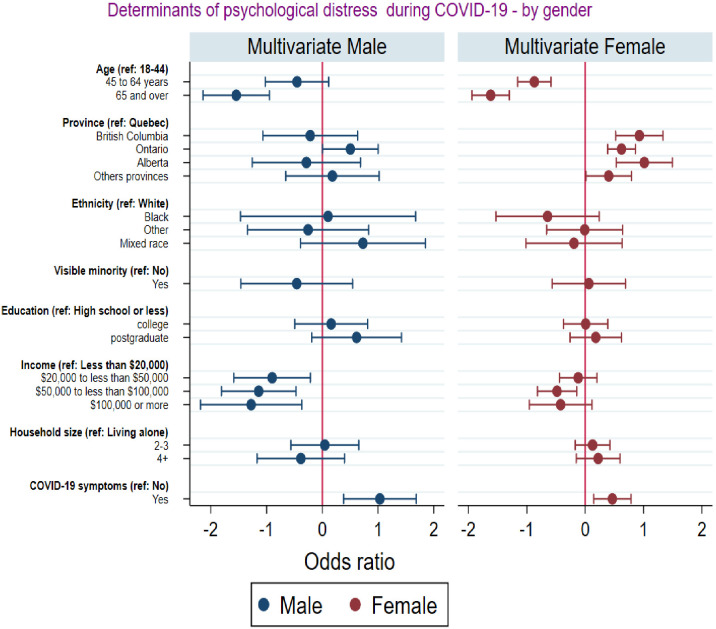
Multinomial logit results on the predictors of psychological distress by gender.

Fig 4 shows the predictors of psychological distress disaggregated by gender. The analysis shows that for both males and females, respondents above 45 years old and those living in Quebec have lower odds of psychological distress compared to respondents aged 18–44 years and those living in provinces other than Quebec, respectively. Interestingly, the magnitude of the point estimates shows that these effects are more pronounced for females relative to males. Similarly, Fig 4 shows that the odds of psychological distress decreases with income for both genders, but the effect is more pronounced and highly significant for males relative to females. Finally Fig 4 also reveals that for both males and females, respondents experiencing COVID-9 symptoms have higher odds of experiencing psychological distress compared to respondents who did not experience COVID-19 symptoms. This latter effect is slightly more pronounced among males compared to females.

## Discussion

Even with the advancement of vaccines to control the COVID-19 pandemic, the disease continues to have social, economic, and health effects on thousands of Canadians. As such, it has become imperative to ensure that this pandemic does not result in yet another and greater health crisis—that of mental illness. Hence, understanding the predictors of poor mental health during the pandemic is critical for the protection and promotion of optimal mental health of the population. We, therefore, examined the predictors of psychological distress among the Canadian adult population in the early period of the COVID-19 pandemic. The descriptive analysis indicates that close to half of the respondents (49%) experienced mild to severe psychological distress.

Our study also revealed the gender disparity in the experience of psychological distress during the pandemic. Compared to males, females had a higher likelihood of reporting psychological distress. This is consistent with the findings from a related study that examined factors associated with psychological distress amidst the COVID-19 pandemic [17]. Previous studies have indicated that during pandemics, environmental strain as well as pre-existing depression and anxieties are heightened among females [18, 19]. Also, the observed association could be explained from the perspective that females are often subjected to violence and abuse, both of which ordinarily result in substantial psychological distress [20]. Our analysis suggests that the COVID-19 pandemic only served as a conduit for exacerbating these pre-existing mental health issues among females.

Analogous to the findings of some related studies [21, 22], we found lower likelihood of experiencing mental health issues as age increased. Thus, older adults were at significantly low risk of mental health issues. As McKinlay, Fancourt, and Burton argue, persons of older age “may have regulated their emotions by focusing on the positive or choosing activities and interactions that reduced their stress” [22]. Consequently, they have less risk of psychological distress such as depression, anxiety, and less interest in doing things. Another explanation for the observed association could be that older adults may have inferred from their previous experiences to comprehend and manage stress related to COVID-19 and its concomitant restrictions [23], thereby reducing their likelihood of feeling nervous, anxious, depressed, not being able to stop worrying or having less interest in doing things. Note, however, that since our survey was a web-based survey, there might be selection bias of those older adults who are active on internet platforms, not institutionalized, and thus might be socially connected and less mentally distressed.

Expectedly, respondents who earned higher income experienced a lower risk of psychosocial distress as compared to those who earned less than $20,000 in 2019. This result is in line with prior evidence showing a lower risk of anxiety and depression among Canadians who earned higher income or were expecting higher income during this period of the pandemic [24]. Our study used data from the first wave of the pandemic in Canada. The first wave of COVID-19 was also the period when non-essential businesses in most provinces were closed alongside lockdowns [6, 7]. Possibly, respondents who earned higher income in 2019 might have accumulated enough money and wealth necessary to meet their economic needs and overcoming the cost associated with the closure of non-essential business and lockdowns imposed by COVID-19. Also, high income earners have better means of protecting themselves from economic shocks and seeking healthcare in need during such situations, which might explain the lower odds of experiencing anxiety and depression among richer Canadians.

Canadians who reported COVID-19 symptoms had higher likelihood of experiencing psychological distress than their counterparts. The result is not surprising, given that the study period coincided with the first wave of COVID-19 in Canada. The first wave of COVID-19 was characterized by abundant uncertainties. Individuals who reported COVID-19 symptoms were mandated to be quarantined and if tested positive for COVID-19, they had to be isolated to prevent further spread of the disease [25]. This resulted in social isolation that exacerbated fears, anxieties, and depression [25–27]. Such actions of social isolation could explain why those who reported COVID-19 symptoms were more likely to experience psychological distress. Our study also revealed that the odds of psychological distress were higher among those who lived in Ontario compared to those who lived in British Columbia. This result could be linked to the already high level of psychological distress among Ontarians as compared to residents of British Columbia prior to the COVID-19 pandemic [25]. Thus, the pandemic and its associated control mechanisms only exacerbated the existing mental health challenge.

In addition, the analysis of the predictors of poor mental health during the pandemic disaggregated by gender suggests that the effects of age, province of residence, and self-reported COVID-19 symptoms on psychological distress were exacerbated among females compared to males. By contrast, the protecting effect of income was more pronounced among males compared to females. These differences might be mirroring the varied gendered roles that males and females perform in our society. Males earn more than females on average. Females are more likely to work in sectors that increase their exposure and vulnerability to pandemics such as nursing, childcare, and front-office jobs. In addition, most females have the responsibility of household chores, taking care of children, and taking care of the sick, which are in themselves physically and mentally burdensome.

### Policy implications

The findings from the present study have some useful implications. First, our findings that Ontarians were at higher risk of experiencing COVID-19 related psychological distress calls on the provincial government to prioritize the mental health of Ontarians just as they ought to prioritize their physical health. Also, our findings underscore the need to target vulnerable groups, that is younger individuals (18–44 years), individuals with lower income, and females in interventions aimed at combating mental illness during pandemics. Our findings also illustrate the urgency for the provincial governments and the department of health to provide continuous counselling to persons who experience COVID-19 symptoms. Finally, our analysis suggests that mental health interventions during pandemics should be gender specific.

### Limitations

This study was successful at determining the determinants of psychological distress during the COVID-19 pandemic among the Canadian population. We applied appropriate weighting which ensured that our data was representative of the national population to increase the generalisation of our findings to the adult Canadian population. Yet, there are limitations that should be considered when interpreting our findings. Our study relied on self-reported data; hence, it is subject to recall bias. We did not include older adults in long-term health care facilities. As such, our findings are limited to the population outside of such institutions. Again, due to the cross-sectional nature of our study design, the effects of the socioeconomic and demographic variables on psychological distress documented in this study may not be causal.

## Conclusion

This study sought to examine the predictors of psychological distress during the COVID-19 pandemic using evidence from a web-based national cross-sectional survey in Canada. Being female, being of lower age, residing in Ontario, experiencing COVID-19 symptoms, and having lower annual income in 2019 were significant predictors of the likelihood of reporting psychological distress. Moreover, most of these effects differed by gender. We conclude that there is a need for Canada to commit substantial resources towards addressing the mental health issues of its most vulnerable population.

## Supporting information

S1 File COVID-19 symptoms & social distancing web survey questionnaire. (PDF)
