# Correction: Psychological distress during the COVID-19 pandemic in Canada

**DOI:** 10.1371/journal.pone.0326593

**Published:** 2025-06-18

**Authors:** The PLOS One Editors

## Notice of Republication

This article [1] was republished on May 16, 2025, to address an issue identified post-publication. An updated version of S2 File is provided with this notice. Please download this article again to view the correct version.

## Supporting information

S2 File Raw data supporting the conclusion of the paper. (DTA)
