# Supplementary material for: Psychological distress during the COVID-19 pandemic in Canada
Source: PLoS One. 2022 Nov 17;17(11):e0277238. doi: 10.1371/journal.pone.0277238 (PMC9671380; doi:10.1371/journal.pone.0277238)
Supplement: S1 File — (PDF) [file pone.0277238.s001.pdf]

Welcome

Project Overview

You are being asked to take part in a panel research survey.

This survey is being conducted to learn about the prevalence of COVID-19 symptoms and social distancing behavior in Canada. We are interested in learning about how education, income, employment, and flu-like symptoms are linked to social distancing and how these links change over time as provincial governments across Canada are implementing lockdown exit policies. The survey also includes questions about your mood and emotional state.

Participation

You are being asked to participate in this survey because you are over 18 years old and are currently living in Canada. Your participation is voluntary, and you may withdraw your participation at any time for any reason. You may skip any question you feel uncomfortable answering.

If you agree to participate in this study, you will be asked to take a short online survey. We will ask you a few questions about COVID-19 symptoms and about your age, gender, income, health, work, and social distancing behaviors. The survey should take less than 10 minutes to complete.

Risks and Benefits

The possible risks of participating in this study include anxiety and stress caused by some of the questions. There is a minimal risk of loss of confidentiality since we do not ask questions that can be used to identify you.

If you decide to withdraw from the study at any time, given the anonymous nature of the study, once the survey is submitted, you will not be able to withdraw your data from the study as the researchers will not be able to retrace the individual datasets.

There are no direct benefits to you if you take part in this survey, but the information you provide will be used to protect and promote the health and the social and economic well-being of Canadian residents.

Confidentiality

Once the survey is completed, data will be stored on password-protected computers at uOttawa. It will also be transferred to study team members' personal computers (which are also password-protected) for analysis.

The data will be stored on SurveyMonkey for 2 years, but they will be held indefinitely by the principal investigator. At the end of the study, the public data will be shared with other researchers upon request for replication.

Participants should print a copy of the consent form to keep in their personal

records.

For more information

If you have any questions about this survey, you can contact the research team at the University of Ottawa :

- Dr. Roland Pongou, Professor at the Faculty of Social Sciences (Department of Economics)
- Dr. Sanni Yaya, Professor at the School of International Development and Global Studies
- Dr. Stéphanie Maltais, Postdoctoral researcher
- Dr. Marie Christelle Mabeu, Postdoctoral researcher

covid19sur@uottawa.ca

This research has been reviewed by the Research Ethics Board of the University of Ottawa (uOttawa REB). If you wish to speak with someone from the uOttawa REB, please contact by phone at 613-562-5387 or by email at ethics@uottawa.ca.

Thank you for your valuable time and participation.

* Are you at least 18 years of age and do you currently live in Canada?

- ◯ Yes
- ◯ No

* Do you consent to participate in this survey?

- ◯ Yes
- ◯ No

Background Questions

We would now like to ask you a few background questions.

In which year were you born?

In which month were you born?

What is your gender?

- Male
- Female
- Other
- Prefer Not to Answer

Are you of a visible minority group?

- Yes
- No
- Prefer Not to Answer

How would you best describe yourself?

Select all that apply

- [ ] Caucasian (White)
- [ ] Black
- [ ] Aboriginal/Indigenous
- [ ] Asian
- [ ] Latin American
- [ ] Arab
- [ ] Other
- [ ] Prefer Not to Answer

In which province or territory in Canada do you currently reside?

What is your postal code?

If you prefer not to answer or do not know, you may skip this question.

What is your highest level of educational attainment?

- ◯ Less than high school
- ◯ High school
- ◯ Some college/CEGEP
- ◯ 2 year associate degree from a college/CEGEP or university
- ◯ 3 or 4 year college/CEGEP or university degree
- ◯ Some postgraduate or professional schooling
- ◯ Postgraduate or professional degree, including master’s, doctorate, medical or law degree
- ◯ Prefer Not to Answer

What was your total individual income in 2019?

- Less than $20,000
- $20,000 to less than $50,000
- $50,000 to less than $100,000
- $100,000 or more
- Don't Know
- Prefer Not to Answer

Do you currently have health insurance?

- Yes
- No
- Don't Know
- Prefer Not to Answer

How many people are currently living in your household?

INCLUDE yourself, other family members, roommates, and temporary visitors

Health

We are now going to ask you some questions about your health.

Have you experienced any of the following symptoms in the past 2 weeks?

Select all that apply

- ☐ Fever
- ☐ Dry Cough
- ☐ Shortness of Breath
- ☐ Decreased Sense of Smell/Taste
- ☐ Other Flu-Like Symptoms
- ☐ None of these
- ☐ Don't know

Have you experienced this symptom in the last 4 days?

Select all that apply

- ☐ Fever
- ☐ Dry Cough
- ☐ Shortness of Breath
- ☐ Decreased Sense of Smell/Taste
- ☐ Other Flu-Like Symptoms
- ☐ None of these
- ☐ Don't know

Do you think any of the symptoms you reported might be related to COVID-19?

- ◯ Yes
- ◯ No
- ◯ Don't know

Has anyone else in your household besides yourself experienced any of the following symptoms in the past 2 weeks?

Select all that apply.

If you live alone, you may skip this question.

- [ ] Fever
- [ ] Dry Cough
- [ ] Shortness of Breath
- [ ] Decreased Sense of Smell/Taste
- [ ] Other Flu-Like Symptoms
- [ ] None of these
- [ ] Don't know

Have you ever been tested for coronavirus (COVID-19)?

- ◯ Yes
- ◯ No
- ◯ Don't know

In which month did you have your most recent coronavirus (COVID-19) test?

What was the result of this coronavirus (COVID-19) test?

- ◯ Positive
- ◯ Negative
- ◯ Don't Know / Inconclusive / Prefer Not To Answer

Did you receive a flu vaccine any time between September 2019 and today?

- ◯ Yes
- ◯ No
- ◯ Don't know

Did anyone else in your household receive a flu vaccine any time between September 2019 and today?

If you live alone, you may skip this question.

- ◯ Yes
- ◯ No
- ◯ Don't know

Do you have any ongoing medical condition that might put you at a higher risk for severe illness from coronavirus (COVID-19)?
Conditions that can put you at a higher risk include:

- Cardiovascular conditions
- Chronic lung disease
- Obesity or diabetes
- A weakened immune system due to smoking, cancer treatment, other immune deficiencies

○ Yes

○ No

○ Don't know

Over the last 2 weeks, approximately how often have you been bothered by the following problems?

	Not at all	Some days	More than half the days	Nearly every day
Feeling nervous, anxious, or on edge	○	○	○	○
Not being able to stop or control worrying	○	○	○	○
Feeling down, depressed, or hopeless	○	○	○	○
Little interest or pleasure in doing things	○	○	○	○

How difficult have these problems you just reported made it for you to do your work, take care of things at home, or get along with other people?

- ◯ Not difficult at all
- ◯ Somewhat difficult
- ◯ Very difficult
- ◯ Extremely difficult
- ◯ Not applicable

Work

We now want to ask you some questions about any paid and unpaid work you might have.

Were you working for pay in February 2020?

- ◯ Yes
- ◯ No

In February 2020, approximately how many people did you come into close contact with during a usual work day (within 6 feet)?
INCLUDE co-workers, customers, students, delivery workers, commuters you travel with, etc.

- ◯ None
- ◯ Less than 10
- ◯ Between 11 and 50
- ◯ Between 51 and 100
- ◯ More than 100
- ◯ Don't Know

Since March 1, 2020, have you seen a decrease in paid income from work?

- ◯ No decrease
- ◯ Some decrease
- ◯ I no longer have any income from work

During the past 7 days, have you done any paid work?

- ◯ Yes
- ◯ No

During the past 7 days, how many days did you leave your home for paid work?

During your most recent day at work, approximately how many people did you come into close contact with (within 6 feet)?
INCLUDE co-workers, customers, students, delivery workers, commuters you travel with, etc.

- ○ None
- ○ Less than 10
- ○ Between 11 and 50
- ○ Between 51 and 100
- ○ More than 100
- ○ Don't Know

What preventive measures have been implemented by your employer to mitigate the risk of Covid-19 spreading in your workplace?

Select all that apply.

- ☐ The wearing of non-medical mask is compulsory
- ☐ The wearing of non-medical mask is highly recommended
- ☐ Hand washing station (with water and soap or with alcohol-based hand sanitizer)
- ☐ Install physical separations between employees/clients (e.g. physical barriers like a plexiglass window or cubicle)
- ☐ Spatial planning ensuring 2 meter separation between employees and/or clients (e.g. moving workstations)
- ☐ Restrict occupant capacity of indoor spaces (Limited number of employees in the workplace)
- ☐ Increase frequency of environmental cleaning, especially of high touch surfaces or equipment
- ☐ Other
- ☐ No specific preventive measures

What personal preventive practices are you following in your workplace to mitigate the risk of Covid-19 spread?

Select all that apply.

- ☐ Wearing a medical or non-medical mask at all times
- ☐ Wearing a medical or non-medical mask occasionally
- ☐ Washing hands frequently with water and soap or with an alcohol-based hand rub
- ☐ Maintaining at least 2 meter (6 feet) distance between yourself and others
- ☐ Cleaning and disinfecting regularly your workstation
- ☐ Other
- ☐ No specific measures

Are you classified as an essential worker?

- ◯ Yes
- ◯ No
- ◯ Don't Know

Are you a health care worker?

- ○ Yes
- ○ No

On a usual day, how many unpaid hours did you spend on care and assistance for family members and friends?
Focus on activities such as:

- Bathing, feeding, teaching, reading, playing, childcare
- Shopping, meal preparation, cleaning, laundry

	Number of hours
February 2020	
Currently	

On a usual day, how many unpaid hours did you spend on volunteer activities?

	Number of hours
February 2020	
Currently	

Prevention & Social Distancing

We would now like to ask you some questions about coronavirus (COVID-19) prevention and social distancing.

Approximately how many times yesterday did you wash your hands with soap and water or use hand sanitizer?

- ◯ 0 times
- ◯ 1 - 6 times
- ◯ More than 6 times

For how long do you usually wash your hands with soap and water each time?

- ○ Less than 10 seconds
- ○ 10 - 19 seconds
- ○ 20 seconds or more

During the past 7 days, how many days did you leave your home?

Did you leave your home yesterday?

- ◯ Yes
- ◯ No

What were your reasons for going out yesterday?
Select all that apply.

- ☐ Work
- ☐ Groceries / shopping / laundromat
- ☐ Bank / ATM
- ☐ Exercise / walk / dog walk / recreation
- ☐ Health care / visit to doctor or pharmacy
- ☐ Meeting friends or relatives
- ☐ Taking out the trash / getting mail
- ☐ Getting tired / bored of being inside of the house
- ☐ Education
- ☐ Religious services / Church
- ☐ Volunteering
- ☐ Other

How many hours were you outside your home yesterday?

Did you cover your face while you were outside your home yesterday?

- ◯ Yes, I wore a face mask (surgical face mask or N95 respirator)
- ◯ Yes, I wore some other face covering (e.g. homemade face covering, cloth covering, scarf)
- ◯ No

How often did you cover your face while you were outside your home yesterday?

- ◯ All the time
- ◯ Some of the time
- ◯ Only when close to other people

Excluding members of your household, how many people in total did you come into close contact with (within 6 feet) yesterday?
INCLUDE any co-workers, relatives, neighbors, delivery workers, other shoppers, etc. that you might have met.

We would like to collect your email address for the survey rounds. Participating in this round of the survey does not oblige you to participate in the next rounds. You may withdraw your participation in this survey at any time. Enter your email address below if you would like us to contact you for the next round of this survey.

End of survey

Thank you again for your time and participation.

Please invite each eligible person in your household to join in the fight against COVID-19 by completing the survey here: https://www.surveymonkey.ca/r/Y2WLWWN

If you or a household member has any of the symptoms that we asked about:

Public Health Agency of Canada has resources and advice on what to do if you or a household member is sick and if you think it may be COVID-19. Follow the link for more details.

If you are experiencing anxiety or stress due to COVID-19:

Crisis Services Canada (1-833-456-4566) is a 24/7, 365-day-a-year, national hotline dedicated to providing immediate crisis counseling for people who are experiencing emotional distress related to any natural or human-caused disaster, including disease outbreaks like COVID-19. This toll-free, multilingual, and confidential crisis support service is available to all residents in Canada and its territories.

If you have any questions about this survey, you can contact the research team at:

covid19sur@uottawa.ca
